# Improving Object Detection in High-Altitude Infrared Thermal Images Using Magnitude-Based Pruning and Non-Maximum Suppression

**DOI:** 10.3390/jimaging11030069

**Published:** 2025-02-24

**Authors:** Yajnaseni Dash, Vinayak Gupta, Ajith Abraham, Swati Chandna

**Affiliations:** 1 School of Artificial Intelligence, Bennett University, Greater Noida 201310, India; 2 School of Computer Science Engineering and Technology, Bennett University, Greater Noida 201310, India; vinayak18dec@gmail.com; 3 School of Artificial Intelligence, Sai University, Chennai 603104, India; ajith.abraham@ieee.org; 4 Department of Information, Applied Data Science and Analytics, SRH University of Applied Sciences, 69123 Heidelberg, Germany

**Keywords:** infrared object detection, object detection, high-altitude detection, thermal imagery, thermal signatures

## Abstract

The advancement of technology has ushered in remote sensing with the adoption of high-altitude infrared thermal object detection to leverage the distinct advantages of high-altitude platforms. These new technologies readily capture the thermal signatures of objects from an elevated point, generally unmanned aerial vehicles or drones, and thus allow for the enhancement of the detection and monitoring of extensive areas. This study explores the application of YOLOv8’s advanced architecture, as well as dynamic magnitude-based pruning techniques paired with non-maximum suppression for high-altitude infrared thermal object detection using UAVs. The current research addresses the complexities of processing high-resolution thermal imagery, where traditional methods fall short. We converted dataset annotations from the COCO and PASCAL VOC formats to YOLO’s required format, enabling efficient model training and inference. The results demonstrate the proposed architecture’s superior speed and accuracy, effectively handling thermal signatures and object detection. Precision–recall metrics indicate robust performance, though some misclassification, particularly for persons, suggests areas for further refinement. This work highlights the advanced architecture of YOLOv8’s potential in enhancing UAV-based thermal imaging applications, paving the way for more effective real-time object detection solutions.

## 1. Introduction

The amalgamation of high-altitude platforms with infrared thermal sensors allows for the generation of sophisticated thermal imagery, which has found its applications in various aspects of monitoring, like surveillance, environmental monitoring, as well as disaster management [1,2]. The fundamental aspect of thermal object detection revolves around the differential emission of thermal radiation by various objects, which allows the sensors to capture the same information. These sensors, mounted on airborne vehicles, allow for the detection of radiation, and based on the thermal properties and ambient temperatures, they paint the resulting thermal images. These resultant thermograms provide a better contrast between the various objects based on the heat signatures, which are differentiable to the human eyes.

Although these images are visually recognizable, the challenge arises in the aspect of unmanned aerial vehicles, which are made to be autonomous to detect and process these thermal images. This prompts the challenge of the processing of high-resolution thermal imagery, as well as the development of robust algorithms for object detection and classification. With all these challenges, the inclusion of advanced machine learning models to improve the accuracy and reliability of object detection became a necessity. The challenge that basic machine learning algorithms face in the aspect of thermal imagery is the presence of a complex, dynamic environment that requires real-time processing. Traditional methods of object recognition are inadequate for the detection of varying object sizes due to occlusion as well as environmental shifts, leading to inconsistent and inaccurate results. The presence of luminance change, shadowing, and perspective distortions further increases the complexity of object recognition tasks [3]. These challenges are particularly pronounced in high-altitude infrared thermal imagery, where thermal signatures can be subtle and easily confused with background noise. The issue of robust feature extraction also hinders the aspect of thermal imagery. The objects that are present in thermal images lack distinct visual features in contrast to the standard features, thus rendering the traditional method of edge detection and texture analysis ineffective. The origin of the images from a high altitude also incorporates atmospheric disturbances and resolution issues, thus exacerbating the challenges. Furthermore, atmospheric disturbances like changing temperatures, wind patterns, and altitude variations can introduce environmental noise into high-altitude imagery. These elements may mask or skew an object’s thermal signatures, making detection even more difficult. Furthermore, it can be challenging to discern targets from the background due to the frequently subtle thermal differences between objects and their surroundings. This is particularly difficult in dynamic environments where abrupt changes in the environment, occlusions, and overlapping objects are common. It must be highlighted that the ambient temperature range typically covers 0 °C to 30 °C, but in terms of the extremities of an environment, it is seen that the temperature ranges from −40 °C to 50 °C in areas like polar regions or arid places. In this temperature range, thermal aspects could be used to highlight objects as the temperature range of a normal human body ranges from 30 °C to 37 °C, which is easily differentiated from the surroundings. Taking the case of objects like machinery and vehicles, it is seen that depending on their operations, these have a temperature range of 30 °C to 100 °C, which sets them apart from the various conditions, thereby concurring with the usage of thermal images as compared to their counterparts to relay information about object detection. 

Another level of complexity is introduced by the variations in the scenarios. For example, because of the differences in thermal emission patterns, detecting objects in open fields versus urban areas may necessitate completely different considerations.

Thus, this sheer amount of data which requires efficiency, accuracy as well as faster processing necessitated the development of sophisticated algorithms for feature extraction and pattern recognition [4]. Convolutional neural networks (CNNs) have been developed to learn and identify the salient features from raw thermal images, thereby increasing the detection accuracy of the objects.

A prominent example of the advancement is the development of the You Only Look Once (YOLO) architecture, which allows for faster processing of images and gives a higher efficacy as compared to its counterparts. This paper thus makes use of the prowess of the YOLO algorithm to implement a resilient and robust object detection algorithm that is suitable for the challenges of thermal images. Although YOLO is advanced, there are certain limitations that are poised when explored in detail. This includes the fact that the extensive parameter space causes YOLO to use more memory space, which slows its inference times. Additionally, it must be highlighted that with increasing complexity, there is also a challenge of sparsity management based on redundant computations, which leads to a decay of efficacy, limiting the feasibility for real-time applications. Furthermore, the absence of dynamic pruning limits the model’s ability to adapt during training, potentially leading to overfitting and the suboptimal generalization of complex datasets. Therefore, owing to this issue, it is important to optimize the usage of YOLO to be more appropriate for this setting. Based on the various advantages as well as the proposal of data preprocessing techniques paired with the usage of YOLO v8, magnitude-based dynamic pruning and non-maximum suppression have been documented in studies to enhance the efficacy of the thermography and image recognition for unmanned aerial vehicles.

The paper is divided as follows: A brief introduction to the issue is presented in Section 1, followed by a survey of the literature and research gaps that have been identified in Section 2. Continuing to Section 3, an overview of the proposed model and the various algorithms is provided; this is followed by the discussions of the results in Section 4, while Section 5 peers into the conclusion of the same topics.

## 2. Literature Review

Object detection in computer vision entails accurately detecting and precisely locating objects within images. Remote sensing images obtained from various sensors offer valuable data for identifying objects on spaceborne, aerial, and ground platforms [5]. Ground remote sensing employs raised structures, vehicles, and ships as platforms for technological systems. Nevertheless, thermal infrared (TIR) datasets have certain limitations. Unmanned aerial vehicles (UAVs) acquire images with high temperature and spatial resolution, thereby overcoming the constraints of satellites. Efficient, intelligent detection techniques are necessary due to the quick progress of UAVs. Precision agriculture uses UAV thermal infrared (TIR) imagery to examine variations in soil and crop conditions, enhancing farming methods and optimizing agricultural inputs [6]. For UAV aerial image object detection, Ref. [7] suggested a novel multiscale feature fusion small object detection network (MFFSODNet). It adds a bidirectional dense feature pyramid network (BDFPN), a multiscale feature extraction module (MSFEM), and an extra micro-object prediction head to decrease parameters and increase detection accuracy. MFFSODNet performs better than state-of-the-art techniques in experiments using the VisDrone and UAVDT benchmark datasets.

Its efficacy is also confirmed on datasets with defects in solar arrays. Unlike optical sensors, TIR sensors can collect images in both day and night settings. Due to the ongoing enhancement of TIR detection system capabilities, TIR technology is now extensively employed in body temperature detection, transportation surveillance systems, and public health and security sectors, garnering significant attention. Nevertheless, there has been limited research on utilizing UAV TIR images and movies for object recognition. Ground thermal infrared pedestrian detection has been explored by [8,9,10] through the application of computer vision and deep learning techniques. The study of ships [11], automobiles [12], thermal bridges in buildings [13], and electrical equipment [14] in TIR images and movies has also been conducted. For instance, Ref. [15] surveyed several applications and surveillance systems that utilize embedded devices to identify human presence in diverse settings. They compared the accuracy and performance time of these systems by employing a unified dataset. The author presented a unique vehicle detection technique utilizing TIR images for the automated monitoring of traffic flow, demonstrating its effectiveness in road traffic surveillance [16]. Deep learning is extensively utilized in image processing and object detection because of its strong ability to discover distinctive features. Convolutional neural networks (CNNs) are the predominant choice for network design [17]. Object recognition systems based on convolutional neural networks (CNNs) can be categorized into two types: two-stage detectors, such as regions with CNN features (RCNN), and one-stage detectors such as You Only Look Once (YOLO), the single shot multiBox detector (SSD), and RetinaNet.

The successful implementations of YOLO have been demonstrated by [18] using an improved development of YOLO-FIRI which is more oriented towards infrared object detection. The application of YOLO-FIRI showed an improvement of 21% in average accuracy as compared to its version 4, encompassing the need for further development in the aspects of YOLO. Furthering the aspects of improvements in YOLO, Ref. [19] compared the usage of the YOLO-based CNN architecture to extract features from the infrared images and conduct object detection. Based on the comparison of the three versions of YOLO comprising 15 variants, the study concluded that YOLOv5 performed well in terms of accuracy and called for better model training for the same model. Furthermore, Ref. [20] explored a novel YOLO and ConvNext-based proposed model of YOLO-CIR for infrared image detection to accommodate the efficacy of high-bit-width infrared images. Their model performed 3% faster than YOLOv5, thus showing that one-shot detectors work well with infrared images, and there is a considerable need for improvement for the same detectors. The implementations of YOLO include the aspect of using Res2Net in conjunction with v8 to enhance feature extraction, but they have shown the fact these implementations lack explanations to computational efficiency and real-time applications [21]. YOLO has also been integrated with optical character recognition (OCR) techniques for license plate detection, which shows that there are more implementations in the area, but they do not contribute to increasing the efficiency and flexibility of the algorithm [22]. By exploring more integrations, it is seen that the context-aware attention mechanism was also included with YOLO to show applications to farming [23]. Although the application is advanced, it is limited with the aspect of efficient resource utilization like sparsity-driven pruning, as shown in our case. In this paper, we used the YOLOv8 model, which has been improved with magnitude-based dynamic pruning and non-maximum suppression to enhance the efficacy and efficiency of thermal object detection by making use of one-stage detectors.

## 3. Materials and Methods

### 3.1. Dataset

**Figure 1:**
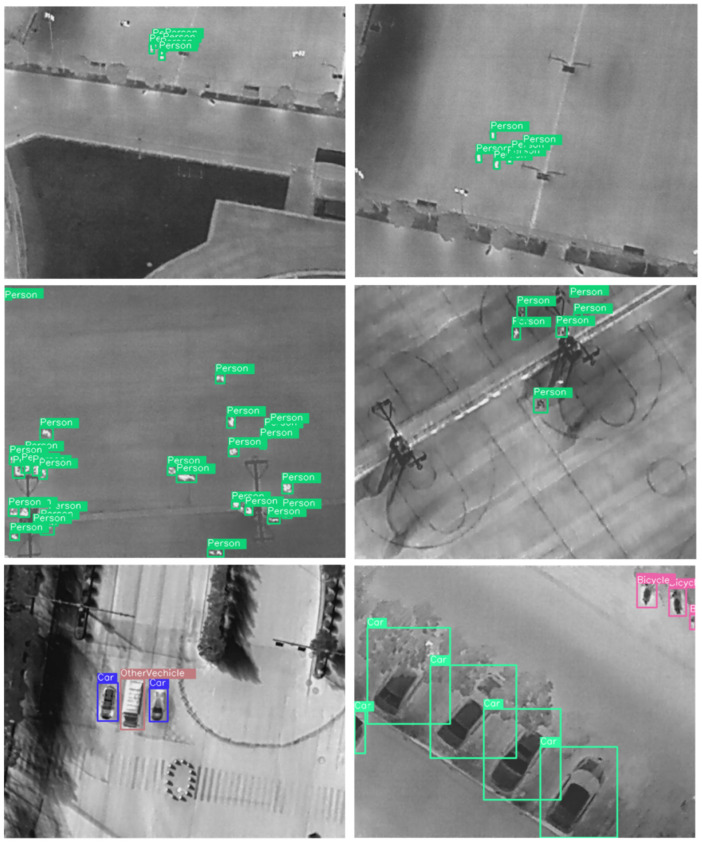
Dataset visualization, with various classes being highlighted.

The dataset used in this research is the HIT-UAV dataset. This dataset is curated for object detection applications about the aspect of unmanned aerial vehicles (UAVs) [24]. The HIT-UAV dataset, consisting of 2898 infrared thermal images, was utilized to evaluate YOLOv8’s performance. The dataset was extracted from 43,470 frames across hundreds of video sequences captured by these UAVs. The dataset spreads over a diverse range of scenarios, which include, but are not limited to, images of schools, parking lots, roads, and playgrounds. This provides a comprehensive foundation for the assessment of the model in different scenarios. The dataset is also diversified in terms of the various aspects of flight metadata, which encompass the factors of fight altitude, camera perspective, as well as daylight intensity. This allows for the better contextualization of thermal imagery and thus helps facilitate better analysis. The dataset, in total, consists of five classes namely the person, car, bicycle, other vehicles, and the dataset does not care about labels, which allows for the utilization of both oriented and standard bounding boxes (Figure 1). This annotation in terms of dual aspects allowed us to address the challenges of object overlapping and thus enhanced the precision of object detection, which allows for a better understanding of the performance of the YOLO architecture.

### 3.2. Model Description

The model used and studied in the research is YOLOv8. YOLO stands for the You Only Look Once (YOLO) family of object detection algorithms, with v8 being the latest addition to it. These sophisticated algorithms allow for the delivery of high-speed, high-accuracy object detection, making it a good choice for complex and dynamic environments, as is the case with the study of high-altitude inferred thermal object detection. The enhancements of YOLOv8 build on the strengths of its predecessors, which employ an improved architecture that allows for the optimization of feature extraction and the utilization of the neural network with more convolutional layers and better residual connections [25]. This allows the model to capture the remote and intricate details of the patterns, which are essential for distinguishing the subtle thermal signatures from high-altitude imagery.

The standout feature of the YOLOv8 is the ability to perform object detection with increased speed and efficacy. This is possible through its aspect of the single-stage detection pipeline, which processes the images in a single pass, as opposed to the traditional architecture, which requires multiple passes to allow for multi-stage detection. The efficacy of the algorithm makes it beneficial for the UAV application, where the promptness of efficient processing will enable it to track objects over larger areas [26]. The presence of anchor-free mechanisms and an adaptive anchor assignment enables architecture to be more robust in terms of the varying object sizes and aspect ratios. This critical adaptability allows for a greater efficacy of high-altitude infrared thermal detection, making the architecture flexible and resilient [27]. The advantage of YOLOv8 includes data augmentation and a more refined loss function; this allows for superior performance under varying environmental noise and varying thermal radiation. These enhancements enable YOLOv8 to achieve higher accuracy and reliability in object detection tasks, addressing the unique challenges posed by high-altitude infrared thermal imagery. By making use of these variabilities, the proposed model aims to improve the accuracy and precision of object detection in high-altitude infrared thermal images, thereby helping all UAV-based applications.

The proposed model of YOLOv8 was enhanced with the help of magnitude-based dynamic pruning. This allows for enhanced efficiency by selectively removing weights or neurons with the smallest magnitudes during training. A pruning method can be found in Algorithm 1 which makes use of sparsity as a parameter to implement the same conditions. Pruning improves efficiency by eliminating unnecessary weights. The inputs include the initial pruning threshold (*τ*initial), target sparsity (*S*target), total epochs (*E*), the pruning interval (*P*), and the decay rate (*α*). During training, weights below a dynamically adjusted threshold are pruned, leading to a sparser network. The pruning is repeated periodically, and the network is fine-tuned to maintain its performance while minimizing unnecessary complexity. This approach reduces model size and computational complexity without significantly losing accuracy. Dynamically adjusting the pruning strategy helps maintain a balance between performance and efficiency, optimizing the model’s resource utilization. Such an approach to the proposed model would allow it to become more refined and robust in terms of training and performance. Further improvement to the robustness of the architecture and the object detection algorithm includes the usage of non-maximum suppression (NMS). NMS is employed to refine object detection results by eliminating redundant bounding boxes. It retains the most confident detection while discarding overlapping boxes, thus reducing false positives and improving detection precision. Together, magnitude-based dynamic pruning and NMS significantly enhance YOLOv8’s performance, making it more efficient and accurate for high-altitude infrared thermal object detection.
**Algorithm 1: Prune Network Weights** 
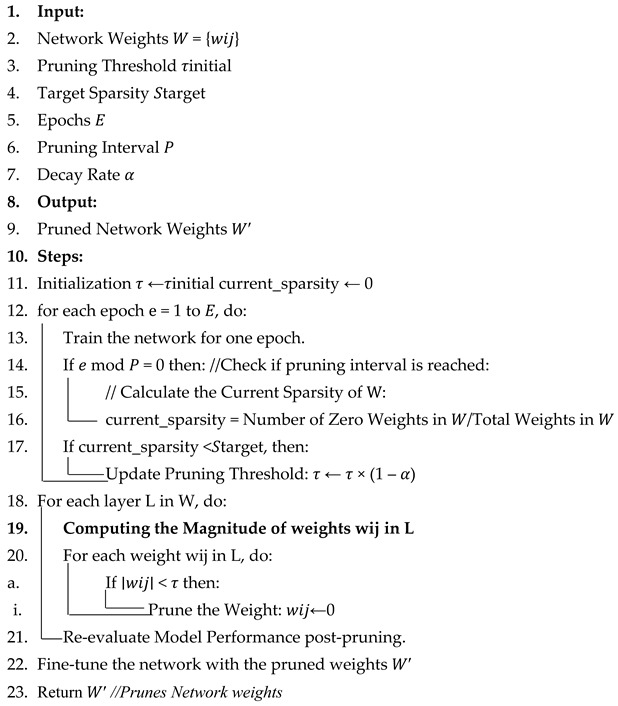


#### 3.2.1. Rationale for the Algorithm

The proposed algorithm makes use of magnitude-based dynamic pruning, which has been rooted in the principle of sparsity-inducing optimization. This method, as the name suggests, is supported by the theoretical background that neural networks are often overparameterized, which surmounts to a significant portion of weights being the negligible contributor to the model’s performance. With the periodic removal of the low-magnitude weights corresponding to less impactful features or even redundant connections, the network can maintain a high level of accuracy without having any extra computational overhead.

The basis of the proposed algorithm is rooted in theory as the pruning follows the principles of sparsity and efficacy in neural network optimization. A cornerstone of the same is the L1-norm sparsity, which is focused on the removal of low-magnitude weights (|*wij*|). Such weights are often attributed to being low in terms of contributions to the output activations and are, thereby, considered less critical to the functionality of the whole network. The alignment of the algorithm with sparsity-inducing norms, like the L1 norm, allows the proposed algorithm to encourage weights to reach zero, which helps in pruning the redundancy of the network as well as the non-essential parameters. This process allows the network to be more effective with a minimum number of redundancies attributed to the same parameters.

The prospect of pruning also allows for the generalization of the network in terms of reducing the complexity of the same network. The various overparameterized networks are usually susceptible to overfitting, with the model being more accustomed to noisy patterns in the training data. The algorithm builds on this aspect via the systematic removal of insignificant weights, thereby helping the model to be simplified and discouraging the aspect of memorization. This enhances the model to be able to generalize the unseen data, and the implied reduction of overfitting allows for better performance on test datasets while maintaining accuracy.

The pruning thresholds were dynamically adjusted during training by incorporating a threshold decay mechanism, allowing the algorithm to adaptively refine the pruning process over time. Initially, a baseline threshold is set, and as training progresses, it decays according to the following formula:

τ = τ × (1 − α)

Here, α is the decay rate, controlling the rate of adjustment. This gradual decay ensures a smoother pruning process by systematically lowering the threshold, enabling the removal of smaller-magnitude weights over time without abruptly impacting model performance. The dynamic adjustment is tied to the pruning interval P, meaning thresholds are recalculated after every P epoch. This stepwise refinement aligns with the model’s convergence, ensuring the pruning remains sensitive to the network’s evolving parameters while maintaining a balance between sparsity and accuracy.

The dynamic adaptation embedded in the algorithm also adds to its effectiveness. The threshold decay mechanism (*τ* ← *τ* × (1 – *α*)) allows for the pruning process to gradually adapt over time and become more sophisticated. This approach ensures that an optimal balance is struck between sparsity and model performance. The algorithm’s gradual lowering of the pruning threshold prevents the abrupt removal of features. This allows the network to retain the essential aspects while also improving computational efficacy.

The ablation study shows how various pruning thresholds affect an algorithm’s performance. The balance between sparsity and model accuracy is directly impacted by threshold selection. While higher thresholds preserve redundant parameters, lower thresholds may eliminate important parameters. To preserve predictive power and reduce the possibility of losing important information, this study recommends a dynamic, iterative method for adjusting thresholds based on the parameter α. This adaptive method improves the accuracy and performance of the model.

#### 3.2.2. Time Complexity Analysis

The time complexity of the proposed pruning algorithm is primarily driven by the training and fine-tuning phases, each requiring O(E⋅n⋅D), where E is the number of epochs, n is the number of weights, and D is the size of the training data. Pruning introduces an additional overhead of O(E/P⋅n), where P is the pruning interval, as it involves calculating sparsity and comparing weights against the threshold. However, this overhead is minimal compared to training and fine-tuning. As pruning reduces n, subsequent training becomes more efficient. Overall, the algorithm balances computational costs and model optimization, ensuring scalability while maintaining accuracy.

#### 3.2.3. Space Complexity Analysis

By analyzing the space complexity of the algorithm, it is therefore highlighted that the storage requirements depend on the network weights and the pruning threshold. At each epoch, it is seen that W weights must be stored, which comprise *m x n* elements that need to be handled, where m stands for many layers and n for the number of neurons. The presence of other variables like the pruning threshold and the sparsity calculations does not necessarily affect the network because they in turn reduce the values of the weight rather than storing them in additional data structures. Therefore, it is seen that for each layer, the space for each computation is *O*(*m x n*), but this does not affect the additional requirements of space storage. The pruning operation iteratively computes the layers without any addition to the weight storage and therefore results in a linear relation to the size of the model.

#### 3.2.4. Novelty of the Algorithm

The proposed algorithm included in this study is adept due to its ability to address the various challenges described in terms of high-altitude thermal imaging and YOLO. The traditional methods are usually limited to the usage of static thresholds that cater to dense networks, making them computationally ineffective. The proposed algorithm, on the other hand, uses dynamic-based pruning mechanisms, allowing for better thresholds based on the sparsity and performance of the model. The dynamic nature of the pruning algorithm allows for a balance between the computational efficiency and accuracy of the model, which is often an important facet in resource-constrained environments like real-time applications operating at higher altitudes.

### 3.3. Data Preprocessing

Preprocessing the dataset is a crucial step of the proposed study to ensure that the data are compatible with the YOLO algorithm. The YOLO algorithm requires data in each format, and thus, the data that were stored in the two widely used formats of COCO (Common Objects in Context) and PASCAL VOC (Visual Object Classes) needed to be handled. These formats have their structure for storing annotation data; for example, the COCO format stores the annotations in the form of JSON files, which contain detailed information about each file and its corresponding objects [28].

**Figure 2:**
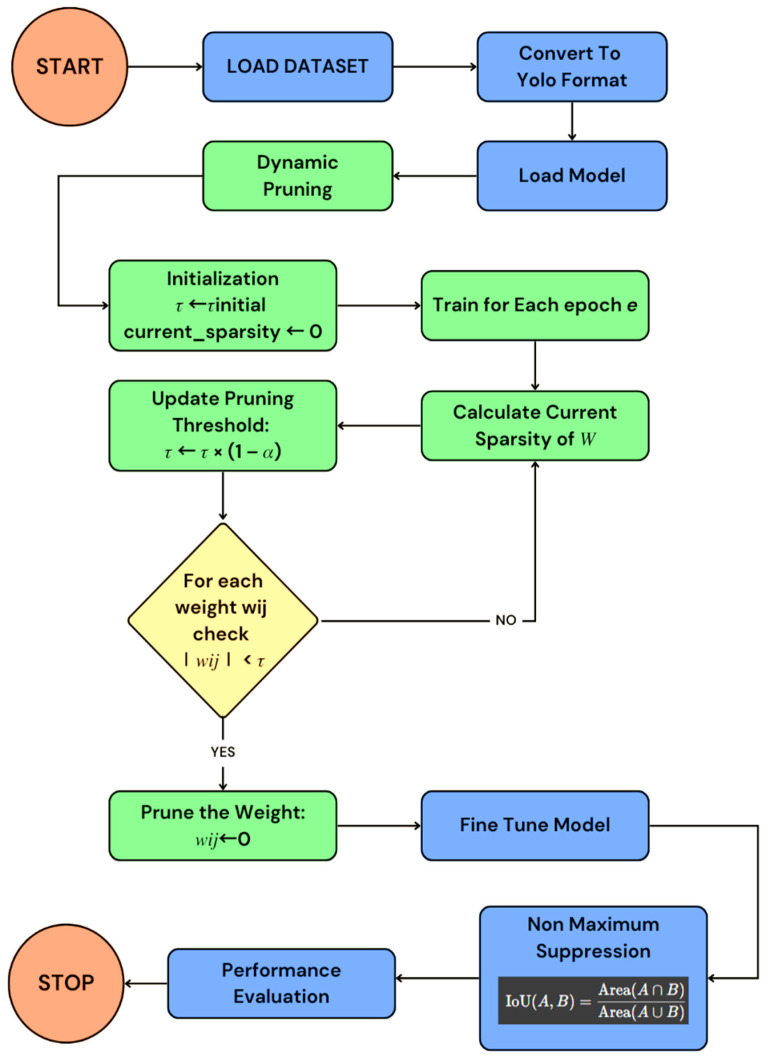
A flowchart of the model workflow showing the various steps in processing.

The bounding boxes in such a case are defined by the coordinate of the top-left corner, with the width and height included. The PASCAL VOC uses XML files, where the bounding boxes are defined by the coordinates of the top-left and the bottom-right corners of each object. Further processing of the whole dataset required the resizing of the images to ensure that there is uniformity for the input dimensions of the proposed algorithm to ensure that the dataset was sufficient for various transformations in terms of rotation; inversion as well as mirroring of the dataset was also performed to increase the instances of the various classes, thereby boosting the learning capacity of the model. A workflow of the model is shown in Figure 2.

## 4. Results

**Figure 3:**
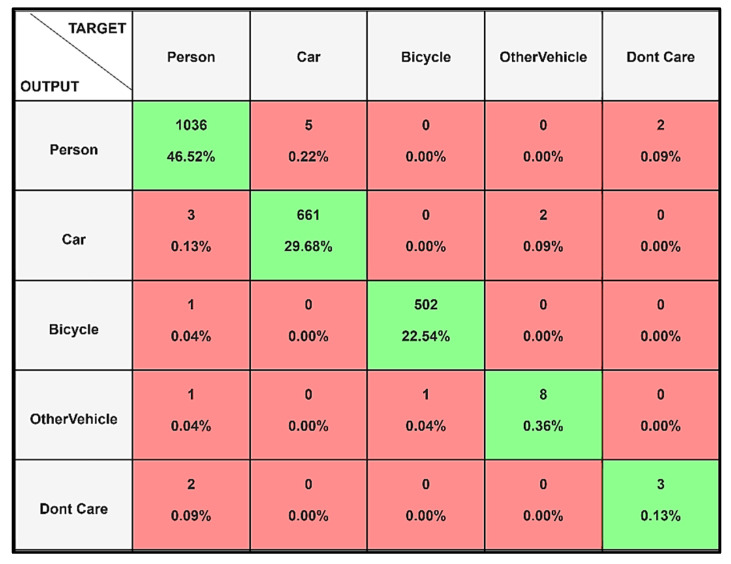
Confusion matrix of the various classes indicating the model performance.

The proposed architecture of the YOLOv8 performed well on the given data with less time for processing, with 25,842,665 parameters being trained over an estimated time of 61.98 min, with an average of 0.3 ms for each preprocess and 6.3 ms for each inference. The training of the model, running for a total of 250 epochs, was performed on the CUDA interface, thus allowing for easier and faster preprocessing of the data. The model’s performance in terms of each class is shown in the confusion matrix in Figure 3. It shows that the model performed well in terms of each of the classes like the person class, car class, and others. To obtain a better understanding of the same model, the model, in terms of its performance to the various listed classes, is analyzed. It shows that the model performed well in the classification of the various classes, but there was still room for improvement for the various underlying classes to understand the computational complexity.

Table 1 shows the model’s performance regarding the relationship between precision and recall for the various accuracy metrics, as well as the F1 score. It shows an accuracy of 0.9924, a small misclassification rate of 0.0076, a micro-weight F1 of 0.8768, and a weighted F1 of 0.9924. The figure illustrates the trade-off between precision (positive predictive value) and recall (sensitivity) across different thresholds, providing insights into a model’s ability to identify relevant instances while minimizing false positives. The results highlight exceptional classification and detection accuracy.

Comparing the model with the original images in terms of the classification and confidence percentage, the figures below show the original label and the prediction of the model. The assessment of the model utilizing precision, recall, and F1-score effectively underscores its overall performance. Nevertheless, it has been noted that specific classes, such as the ‘Don’t Care’ category, exhibit a decrease in detection accuracy relative to other categories like ‘Person’ or ‘Car’. This discrepancy can be ascribed to the inadequate instances of the ‘Don’t Care’ class, which probably resulted in insufficient representation during the training phase. The inherently ambiguous nature of this class complicates the model’s ability to establish precise boundaries and attain elevated confidence levels during classification.

This trade-off signifies that although the model proficiently identifies prominent and well-represented classes, it struggles with underrepresented or less distinct categories. Resolving this issue may require the implementation of data augmentation techniques specifically aimed at the ‘Don’t Care’ class or modifying the loss function to more effectively penalize misclassifications for these categories. Implementing these strategies may enhance the model’s equitable performance across all classes while maintaining overall accuracy.

**Figure 4:**
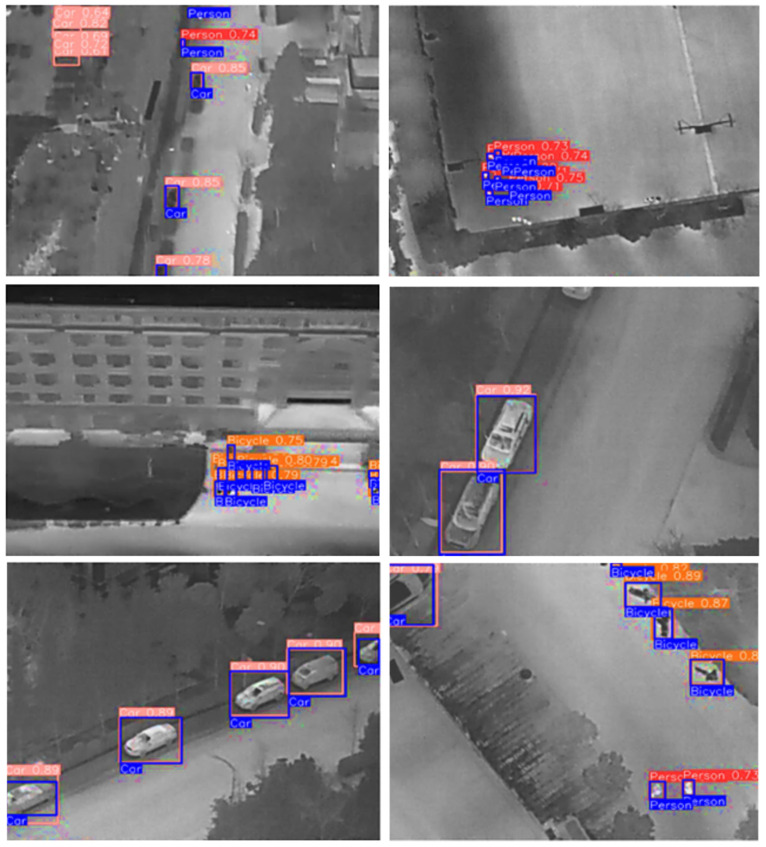
Original classes and model predictions indicate the prediction probability with the bounding boxes.

From Figure 4, we see that the model fared well in terms of the classification of the car and the person class, and the confidence ratio of the same class is also higher. This in turn depicts that the model is working well in terms of the classification and identification of objects in the dataset for the application of thermal image identification.

By gauging the performance of the model, it was also important to perform an ablation study to highlight the performance of the various components as well as to highlight the need for the given study as compared to the various models in terms of the other state-of-the-art models that have already been implemented. An overview of the same can be found in Table 2. The results highlight exceptional classification and detection accuracy.

From the results in the table, the performance of the model as compared to the various state-of-the-art models as well as the successful implementations of YOLO could be seen in the performance of the model in terms of proving its effectiveness compared to the base model of YOLOv8 as well as its counterparts as suggested by the various studies, showing that it works better in terms of giving higher accuracy in real time.

### 4.1. Benefits of the Proposed Algorithm

The proposed system offers transformative potential in ecology and biodiversity research, particularly in tracking and predicting object dynamics and movement patterns. Beyond mere detection, it enables the precise visual tracking of animals, offering insights into their behavioral patterns, migration routes, and interactions. Unlike GPS-based systems, which are costly, require physical tagging, and often face signal reliability issues, this system leverages high-altitude thermal imaging to non-invasively monitor animals over large areas. This approach reduces costs, minimizes ecological disturbance, and enhances data collection in remote or harsh terrains, such as dense forests, arid deserts, or polar regions. The algorithm’s adaptability allows it to predict motion patterns, which is invaluable for understanding population dynamics, identifying habitat usage, and mitigating human-wildlife conflicts [29]. For instance, tracking animal movements near highways can help prevent accidents, while monitoring migratory birds supports conservation planning. The system’s scalability ensures that it can manage large datasets, making it suitable for tracking herds or flocks over extended periods. Additionally, the system’s robustness enables it to operate effectively across diverse environmental conditions. Its capacity for dynamic adaptation, such as adjusting to thermal variations, ensures reliable performance in varying terrains and climatic conditions. These capabilities make it a valuable tool for movement ecology and biodiversity studies, enhancing our ability to monitor ecosystems at scale. By integrating visual tracking, the system extends its utility beyond ecology to applications like traffic monitoring, offering cost-effective, scalable solutions for tracking vehicles and predicting congestion dynamics in urban settings.

### 4.2. Ethical Considerations

The proposed algorithm has applications catering to both military applications and civil applications, which include the aspects of tracking vehicles, wildlife monitoring, as well as individuals in a high-altitude setting. The ethical considerations that arise in this aspect include the various aspects of development and deployment in user settings. This project calls for better standards of transparency by adhering to international guidelines on the ethical use of surveillance and object detection systems. The algorithms prioritize civil applications, such as wildlife monitoring, disaster management, and environmental research, over military applications.

### 4.3. Limitations

Environmental factors like heavy fog or rain introduce noise and occlusions in images, which can severely impact the detection and classification accuracy of object detection models like YOLOv8. These situations call for strong feature extraction skills and modifications to the model’s training procedure. In theory, by exposing the model to a variety of situations and adding extra data augmentation methods like simulating fog or rain effects during preprocessing, we can improve the model’s resilience. The domain adaptation principle, which teaches models to generalize across a range of environmental conditions, is in line with this strategy. Johansen et al. [30] explored how weather conditions influence object detection in thermal imagery. They proposed a novel approach to estimate weather parameters, aiming to improve detection accuracy under varying conditions. Bao et al. [31] introduced the dual-YOLO framework, which integrates data from both infrared and visible imagery to enhance object detection capabilities. Kim et al. [32] addressed the challenge of detecting small objects in infrared images by using domain adaptation techniques to mitigate the issue of cross-domain data imbalance. Similarly, Xi et al. [33] developed IRSDet, a neural network architecture featuring sparse-skip connections and guide maps, specifically tailored for improving the detection of small objects in infrared imagery.

Moreover, transfer learning can further aid in addressing the issue of underrepresented classes. By leveraging pre-trained models trained on extensive datasets, the network can gain a better initial understanding of features, even for less prevalent categories. Fine-tuning these models on specific datasets ensures that underrepresented classes receive focused attention, improving the model’s overall performance and adaptability in real-world applications. These strategies collectively enhance the resilience of object detection systems to adverse environmental conditions while ensuring the equitable representation of all classes.

### 4.4. Contribution to Energy Efficiency with Sustainability Objective

Pruning involves the computation of sparsity and the comparison of weights against the threshold. However, it has a minimal overhead O(E/P⋅n) as compared to training and fine-tuning. The training becomes more efficient as pruning reduces n. Hence, the proposed pruning algorithm theoretically contributes to energy efficiency by reducing the computational complexity of the model, as fewer parameters require fewer operations during both training and inference. This reduction in operations can lead to a decrease in power consumption, which is particularly relevant for UAV-based applications, where energy resources are limited. By systematically eliminating low-magnitude weights, the algorithm optimizes resource utilization, potentially lowering the demand for GPU or CPU cycles and, in turn, reducing the energy footprint.

Furthermore, dynamic pruning through a threshold decay mechanism ensures that the trade-off between sparsity and performance is carefully managed, preventing unnecessary computational overhead. Aligning these optimizations with sparsity principles indirectly supports sustainability goals by enabling UAV systems to perform real-time processing tasks with reduced energy consumption and prolonged operational capacity. Future studies could enhance this perspective by quantitatively evaluating the energy savings achieved, which would offer a concrete measure of the model’s alignment with sustainability objectives.

## 5. Conclusions

The integration of YOLOv8 with the magnitude-based dynamic pruning and non-maximum suppression into high-altitude infrared thermal object detection presents a significant advancement in addressing the complexities of remote sensing. The YOLOv8 architecture, with its refined feature extraction and processing capabilities, has demonstrated considerable effectiveness in managing the challenges inherent to high-resolution thermal imagery. The model’s ability to perform real-time object detection with remarkable speed and accuracy underscores its suitability for UAV-based applications, where timely and reliable object recognition is crucial. The preprocessing of data, transitioning from the COCO and PASCAL VOC formats to YOLO-compatible annotations, has facilitated a seamless adaptation of the dataset for YOLOv8. This conversion has ensured that the model operates efficiently, leveraging the normalized bounding box coordinates to enhance object detection precision. The results highlight YOLOv8’s adeptness at handling the dynamic and variable conditions of high-altitude thermal images, effectively managing overlapping objects and varying thermal signatures. The performance metrics, including training efficiency and precision–recall ratios, reflect the model’s robust capability in distinguishing between different classes with minimal misclassification. Notably, the model’s high confidence levels in classifying objects like cars and people demonstrate its reliability in real-world applications. However, the observed misclassification rates suggest areas for further refinement, particularly in enhancing the model’s sensitivity to subtle thermal features. The dynamic pruning mechanism guarantees adaptability to real-time data streams, where computational limitations necessitate models that are both efficient and effective. Furthermore, the model’s exceptional accuracy metrics, demonstrated by a precision of 0.993 for the ‘person’ class, underscore its reliability in crucial detection situations, including the identification of individuals in crowded or high-risk settings. The model’s exceptional accuracy and computational efficiency render it ideal for tasks such as overseeing high-traffic areas or improving safety in restricted zones. These applications correspond with the model’s proven proficiency in managing various object categories with exceptional precision. These factors substantiate the recommendation to incorporate the model into surveillance systems, thus aligning the research with practical requirements while demonstrating its theoretical and practical importance. The proposed architecture has demonstrated efficacy in enhancing thermal image recognition and facilitating superior UAV-based surveillance and monitoring solutions. Future research may concentrate on rectifying the identified limitations and enhancing the model’s performance to maximize its potential in varied and demanding environments.

## Figures and Tables

**Table 1:** Model metrics across various classes and parameters.

Class Name	Precision	Recall	F1 Score	mAP
**Person**	0.9933	0.9918	0.9956	0.9739
**Car**	0.9925	0.9988	0.9915	0.9607
**Bicycle**	0.9890	0.9888	0.9842	0.9467
**Other vehicle**	0.8000	0.8548	0.8942	0.9652
**Don’t Care**	0.6000	0.6943	0.6183	0.6687

**Table 2:** Comparison results of the proposed model with benchmarked models.

Model	Accuracy
YOLO-FIRI [18]	0.762
YOLOv3-spp [19]	0.909
YOLOv4-pacsp-mish [19]	0.853
YOLOv5-s [19]	0.918
YOLOv8	0.878
YOLOv8-n	0.923
YOLOv8-m	0.917
YOLOv8-s	0.953
YOLOv8-l	0.895
YOLOv8-x	0.934
Proposed model (YOLOv8 + Dynamic Pruning)	0.993

## Data Availability

The data is freely available on the Kaggle network.
