# Correction: Dash et al. Improving Object Detection in High-Altitude Infrared Thermal Images Using Magnitude-Based Pruning and Non-Maximum Suppression. *J. Imaging* 2025, *11*, 69

**DOI:** 10.3390/jimaging12080371

**Published:** 2026-08-11

**Authors:** Yajnaseni Dash, Vinayak Gupta, Ajith Abraham, Swati Chandna

**Affiliations:** 1 School of Artificial Intelligence, Bennett University, Greater Noida 201310, India; 2 School of Computer Science Engineering and Technology, Bennett University, Greater Noida 201310, India; vinayak18dec@gmail.com; 3 School of Artificial Intelligence, Sai University, Chennai 603104, India; ajith.abraham@ieee.org; 4 Department of Information, Applied Data Science and Analytics, SRH University of Applied Sciences, 69123 Heidelberg, Germany

## Error in Table

In the original publication [1], there was a mistake in Table 1 as published. In Table 1, the F1-score values were inadvertently misreported due to a formatting/transcription error during manuscript preparation. The correct F1 values calculated using the standard harmonic mean formula are provided below.

F1 = 2PR/(P + R)

The corrected Table 1 appears below.

## Text Correction

In the second paragraph of the Results section, the term “a micro-weight F1 of 0.8768” should be read as: “a micro-weight F1 of 0.8895”.

The macro-F1 value has also been recomputed to ensure consistency with the corrected class-wise F1 scores and confusion matrix statistics. This correction clarifies the intended evaluation metric used for summarizing class-wise performance across the object categories.

These corrections relate only to the presentation of evaluation metrics and do not affect the experimental setup, methodology, result interpretation, or conclusions of the study. The confusion matrix, accuracy value (0.9924), and other reported performance metrics remain unchanged and internally consistent.

The authors state that the scientific conclusions are unaffected. This correction was approved by the Academic Editor. The original publication has also been updated.

## Figures and Tables

**Table 1:** Model metrics across various classes and parameters.

Class Name	Precision	Recall	F1 Score	mAP
Person	0.9933	0.9918	0.9926	0.9739
Car	0.9925	0.9988	0.9956	0.9607
Bicycle	0.9890	0.9888	0.9889	0.9467
Other Vehicle	0.8000	0.8548	0.8265	0.9652
Don’t Care	0.6000	0.6943	0.6437	0.6687
